# 3D Printing of Clear Orthodontic Aligners: Where We Are and Where We Are Going

**DOI:** 10.3390/ma13225204

**Published:** 2020-11-18

**Authors:** Cinzia Maspero, Gianluca Martino Tartaglia

**Affiliations:** 1 Department of Biomedical, Surgical and Dental Sciences, School of Dentistry, University of Milan, 20100 Milan, Italy; gmtartaglia@gmail.com; 2 Fondazione IRCCS Cà Granda, Ospedale Maggiore Policlinico, 20100 Milan, Italy

## Editorial

Clear orthodontic aligners were developed at the end of the 1990s [1]. The primary benefits are the improved esthetics and comfort, and the possibility to remove them when eating, brushing and flossing [2,3]. Different thermoplastic materials, or their combinations, are used for thermoforming clear aligners, including polyvinyl chloride, polyurethane, polyethylene terephthalate, and polyethylene terephthalate glycol [4,5,6]. However, a physical model (produced by 3D printing, stereolitography or material jetting) is needed for each aligner to be thermoformed and finally trimmed [7,8,9]. In addition, significant changes of material properties due to the thermoforming process have been reported. Ryu et al. [10] showed that thermoforming decreases the transparency of thicker material, and increases water absorption, water solubility and the surface hardness of the tested materials. While increased the higher thickness increases the delivered force and decreases the flexure modulus, the thermoforming process decreases both properties. The regular thickness of the aligner plays an important role in the magnitude of delivered forces: irregularities in the thickness influences the fitting accuracy of the aligner [7].

Moreover, other studies showed that temperature, humidity and salivary enzymes affect the aligner, modifying its original shape and mechanical behavior [11,12,13,14]. The described changes of the aligner caused by thermoforming process and intraoral environment then might influence the treatment efficacy. An alternative to the described conventional fabrication is now approaching the direct 3D printing of clear aligners using dedicated materials [15].

The 3D printing technologies are currently increasing their role for clinical and research purposes in dentistry. Surgical implant guides, prosthodontics, restorative dentistry, orthodontics, implantology and instrument manufacturing have been revolutionized by 3D printing [15,16,17]. This technology allows to manufacture components layer-by-layer, instead of common manufacturing methods that rely on molding, machining or other subtractive methods [18,19]. Acrylonitrile-butadiene-styrene plastic, stereolithography materials (epoxy resins), polylactic acid, polyamide (nylon), glass-filled polyamide, silver, steel, titanium, photopolymers, wax, and polycarbonate are commonly used materials for 3D printing in orthodontics [20,21,22,23,24].

This technology can be used for direct printing of clear aligners, too [15]. Despite today’s improvement of 3D printing technology, the lowering of its cost and its use in other fields of dentistry, a limited number of studies can be found describing the direct 3D printing of clear aligners.

Multiple 3D printing processes may be used for the direct printing of clear aligners, such as fused deposition modelling, selective laser sintering, selective laser melting, direct pellets fused deposition, stereolithography, multi-jet photo cured polymer process, or continuous liquid interface production technology [15,25,26,27]. However, 3D printing by photo-polymerization from clear resin is currently the most suitable option.

A study by Jindal et al. [28] presented a successfully 3D printed 0.75 mm thick clear aligner using Dental Long Term (LT)^®^ clear resin (Form Labs, Somerville, MA, USA) and compared its mechanical and geometrical properties to a conventionally manufactured thermoformed Duran clear dental aligners. Authors suggested that 3D printed resin-cured clear dental aligners are more suitable because they have better geometric accuracy and mechanical resistance. Dental LT resin is an approved Class IIa biocompatible material with high resistance to fracture and it is ideal for hard splints, retainers, and other rigid direct-printed long-term orthodontic appliances. Its use for a direct 3D printing of clear aligner was not described before, and authors are omitting the major limitation of their study–the lack of clinical data to evaluate performance of Dental LT resin and its durability during the use by patients.

The cytotoxicity of directly 3D printed clear aligners from three different materials was also studied [29]. Three different aligners were compared: printed using Accura 60^®^ SLA (3D Systems, Rockhill, South Carolina), Dental LT^®^ clear resin (Form Labs, Somerville, MA, USA) and Invisalign^®^ (Aligntech, San Jose, CA, USA). Similarly, Dental LT resin and Accura 60^®^ SLA are not approved for direct printing of 3D aligners and were not previously tested and described for this purpose. The study confirmed that Invisalign^®^ material was the least cytotoxic, followed by Dental LT^®^ and Accura 60^®^. However, to our knowledge, Invisalign^®^ aligners are not directly 3D printed. The lack of clinical data is similarly the main limitation of this study.

In early 2018, EnvisionTEC Inc. announced launch of E-Ortholign, a new material for the direct 3D printing of clear thermoformed aligners [27,30,31,32]. This clear resin is marketed as biocompatible, stable, flexible and strong material for the direct 3D printing of esthetic retainers [30,33,34]. Nowadays, there is no marketed and approved resin suitable for the direct 3D printing of clear aligners.

Direct 3D printing of aligners offers several advantages over conventional fabrication: edges are softer and do not need trimming or smoothing; undercuts analysis is digitally defined; intra-aligner thickness is customizable, which may reduce the need of attachments, reducing the transparency of clear aligners [35].
